# Measuring Food-Related Attentional Bias

**DOI:** 10.3389/fpsyg.2021.629115

**Published:** 2021-10-13

**Authors:** Stefania Franja, Anna E. McCrae, Tina Jahnel, Ashley N. Gearhardt, Stuart G. Ferguson

**Affiliations:** ^1^College of Health and Medicine, University of Tasmania, Hobart, TAS, Australia; ^2^Institute for Public Health and Nursing, University of Bremen, Bremen, Germany; ^3^Psychology Department, University of Michigan, Ann Arbour, MI, United States

**Keywords:** attentional bias, visual probe, lexical, reaction time, reliability, pictorial

## Abstract

**Objective:** Food-related attentional bias has been defined as the tendency to give preferential attention to food-related stimuli. Attentional bias is of interest as studies have found that increased attentional bias is associated with obesity; others, however, have not. A possible reason for mixed results may be that there is no agreed upon measure of attentional bias: studies differ in both measurement and scoring of attentional bias. Additionally, little is known about the stability of attentional bias over time. The present study aims to compare attentional bias measures generated from commonly used attentional bias tasks and scoring protocols, and to test re-test reliability.

**Methods:** As part of a larger study, 69 participants (67% female) completed two food-related visual probe tasks at baseline: lexical (words as stimuli), and pictorial (pictures as stimuli). Reaction time bias scores (attentional bias scores) for each task were calculated in three different ways: by subtracting the reaction times for the trials where probes replaced (1) neutral stimuli from the trials where the probes replaced all food stimuli, (2) neutral stimuli from the trials where probes replaced high caloric food stimuli, and (3) neutral stimuli from low caloric food stimuli. This resulted in three separate attentional bias scores for each task. These reaction time results were then correlated. The pictorial visual probe task was administered a second time 14-days later to assess test-retest reliability.

**Results:** Regardless of the scoring use, lexical attentional bias scores were minimal, suggesting minimal attentional bias. Pictorial task attentional bias scores were larger, suggesting greater attentional bias. The correlation between the various scores was relatively small (*r* = 0.13–0.20). Similarly, test-retest reliability for the pictorial task was poor regardless of how the test was scored (*r* = 0.20–0.41).

**Conclusion:** These results suggest that at least some of the variation in findings across attentional bias studies could be due to differences in the way that attentional bias is measured. Future research may benefit from either combining eye-tracking measurements in addition to reaction times.

## Introduction

Attentional bias is the tendency to give preferential attention to stimuli which are personally, motivationally and emotionally relevant (Kuckertz and Amir, 2015). It is of interest to researchers because both theoretical predictions and observational data link attentional bias to important behavioral outcomes, such as food choice/intake (for example, see; Nijs et al., 2010b; van Ens et al., 2019). Attentional bias is commonly assessed using the visual probe task, which was originally developed to study anxiety (MacLeod et al., 1986) and then later used in the addiction field (Mogg et al., 2003). However, this task has increasingly been used to study the link between attentional bias toward food cues and outcomes such as eating patterns and obesity (Field et al., 2016).

Although attentional bias for food has been linked to obesity (Castellanos et al., 2009; Calitri et al., 2010; Nijs et al., 2010b; Kakoschke et al., 2014), numerous studies have reported no such association (Loeber et al., 2012; Garcia-Garcia et al., 2013; Werthmann et al., 2014). It has been suggested that the conflicting findings may be due—at least in part—to differences in the way that attentional bias is assessed from study to study (Nijs and Franken, 2012). For example, various tasks have been used to investigate food-related attentional bias. Tasks such as the Dot Probe (Kemps et al., 2014; van Ens et al., 2019), the Stroop (Nijs et al., 2010a; Phelan et al., 2011), the Flicker (Favieri et al., 2020) and the Go/no-go (Love et al., 2020) share the same underlying goal of assessing food-related attentional bias, but differ in how this is operationalized. Furthermore, variability exists even within-task. For example, the commonly used visual probe paradigm is sometimes conducted with word pairs (Calitri et al., 2010; Kemps et al., 2014) but other times picture pairs are used (Werthmann et al., 2014; Meule and Platte, 2016). Currently, there is no evidence as to whether bias scores obtained from the lexical and pictorial tasks are comparable on an individual level. Adding further variability to the testing procedure, how individual attentional bias tasks are scored also varies across studies. With the visual probe task, a reaction time bias score is generally expressed as a difference score by subtracting the reaction times for the trials where probes replaced neutral stimuli from the trials where the probes replaced target (e.g., food-related) stimuli (van Ens et al., 2019). As such, positive scores indicate a bias toward food related stimuli. However, some studies report bias scores as the difference between; (i) neutral and food stimuli (Ruddock et al., 2018; Fang et al., 2019; Liu et al., 2019; Mas et al., 2019), (ii) neutral and high caloric food stimuli (Favieri et al., 2020; Love et al., 2020), (iii) neutral and low caloric food stimuli (Favieri et al., 2020; Love et al., 2020), or the difference between (iv) low caloric food and high caloric food stimuli (Meule and Platte, 2016; Zhang et al., 2018). It is currently unclear whether these different scoring approaches produce comparable bias scores; as such, we do not know whether these differences in scoring add to the variability in the attentional bias literature. Regardless of the scoring procedure or task used, the results remain varied; findings are inconsistent irrespective of whether studies implement the same procedure or not.

While various tasks have been used to assess attentional bias, the visual probe task is considered “gold standard” by some researchers, remaining the most extensively used in attentional bias research (Kappenman et al., 2014). However, the task’s test-retest reliability has been called into question: studies of attentional bias in the addiction and anxiety fields, for example, have reported poor test-retest reliability (Schmukle, 2005; Christiansen et al., 2015; MacLeod et al., 2019). When examining the test-retest of the visual probe for food-related attentional bias, van Ens et al. (2019) reported acceptable test-retest reliability for all reaction time indices. However, van Ens et al. (2019) used a longer stimulus presentation time than is commonly used in attentional bias studies (stimulus presentation time of 3,000 ms vs. the more standard 500 ms). Little is known about the test-retest reliability of the food-related visual probe tasks when using the presentation time of 500 ms. As such, while theorists generally assume that attentional bias is a more or less static variable, with little variation from moment to moment (for example, incentive sensitization theory; Robinson and Berridge, 1993), the test-retest reliability of our most commonly used task has not been extensively studied.

Attentional bias is of theoretical interest to obesity researchers, but to advance the field researchers must understand more about the reliability of the tests being used. Without consistency across measures, it is possible that the observed differences across studies could be due to differences in the way that attentional bias is assessed from study to study. The aim of the present study was to assess the reliability of the visual probe task. As such, the present study was conducted: (i) to compare attentional bias scores obtained from the lexical and pictorial version of the visual probe task using the different methods of scoring, and (ii) to assess the test-retest reliability of the pictorial probe task. We chose to examine the visual probe task as it is the most commonly implemented task assessing food-related attentional bias. Given the exploratory nature of this study, no specific hypotheses were tested.

## Materials and Methods

### Overview

Data for this study are drawing from a larger study designed to investigate the relationship between attentional bias, impulsivity, and real-world eating patterns^1^. The full data set and the code book can be accessed here: https://rdp.utas.edu.au/metadata/2c3122be-fc62-48d0-a42d-41875d21e71b. In addition to completing a series of laboratory attentional bias tasks—the focus of this paper—, participants in the larger study were also required to track their eating and drinking using a smartphone application during a 2-week real-world monitoring period (similar to that described in Schüz et al., 2015). The results from this field-based monitoring will be reported elsewhere. The study was approved by the Tasmanian Social Sciences Human Research Ethics Committee prior to the first participant being enrolled (H0018038).

### Participants

Potential participants were recruited through a mixture of social media advertisements (see: Frandsen et al., 2013) and flyers placed near the study site. Advertisements called for individuals interested in participating in a study examining eating patterns. Eligibility criteria included being aged 18–75, having no history of eating disorders, not currently dieting, and having a body mass index (BMI) > 18.5. Individuals with concerns regarding body weight, shape and/or eating (as measured by a score of > 20 on the Eating Attitudes Test; Garner and Garfinkel, 1979) were excluded and referred to their general practitioner. We used recruitment targets to ensure that the final sample contained approximately equal numbers of participants in the healthy-weight and high-BMI range. The final sample consisted of 69 participants (67% female) aged 18–71 (*M* = 30.67, *SD* = 11.71), of whom 35% were in the healthy weight range (BMI = 18.5–24.9), 29% in the overweight range (BMI = 25.0–29.9), and 36% in the obese range (BMI > 30).

### Procedure

The full procedure for the present study has been described elsewhere (Franja et al., 2020) and mirrored the protocol used in earlier studies (Elliston et al., 2016). Briefly, after recording their age, anthropometric measurements and contact details in an online portal, eligible participants were invited to visit the lab and received information about the study and provided consent. In line with previous research (Kemps et al., 2014; Werthmann et al., 2014), participants were instructed to eat a light meal up to 2 h prior coming into the lab visits to ensure they were satiated upon arrival. Hunger was assessed at the beginning of study visits using a 100-point hunger scale (Castellanos et al., 2009; Loeber et al., 2012).

During this initial study visit, participants were asked to complete the pictorial probe task followed by the lexical probe task (described in greater detail below). Participants were then issued with a study-specific electronic diary for a field-based monitoring component of the study (data not reported here). Approximately 14 days after this initial session, participants returned to the lab and completed the pictorial probe task for a second time. Tasks at both sessions were completed seated approximately 50 cm in front of a 21.5 inch monitor using Inqusit 5 (Inquisit 5, 2016). Participants were individually tested in two single sessions of approximately 30 min in duration in a well-lit room in the University of Tasmania’s Clinical Research Facility^2^. After testing, participants were thanked and reimbursed AU$60 for their time.

### Materials

#### Lexical Probe Task

The task consisted of 20 food words and 60 animal words. Food words included both high-caloric (e.g., hamburger, brownie), and low-caloric (e.g., broccoli, apple) words. Animal words were made up of species generally not consumed in Western cultures (e.g., cat, hamster). The critical trials were made up of 20 food words paired with animal words, whilst the control trials were made up of animal words paired with other animal words. Based on previous research using this task (Kemps et al., 2014), all word pairs were matched for ratings of valence and arousal, as well as the number of letters and syllables. In addition to the critical (food—animal) and control (animal—animal) trials, an additional 14 word-pairs consisting of stationery items (e.g., pencil, stapler) were used for practice and buffer trials. Participants were asked to place their left index finger on the “T” key and their right index finger on the “B” key. Each trial began with a fixation cross presented in the center of the screen for 500 ms. Following this, word pairs were presented for 500 ms. All words were presented centrally, one above the other, black Arial on a white background, in lower case. After the word presentation, a visual probe (“X”) replaced one of the previously presented words (i.e., either top or bottom). Participants were asked to indicate as quickly as possible (by hitting the relevant keys) which word the probe replaced (top or bottom). The probe remained on the screen until a response was made. The intertrial interval was 500 ms. The whole task consisted of 12 practice, 2 buffer, and 160 experimental trials. During the experimental trials, each of the critical (food—animal) and control (animal—animal) trials were presented four times, at each of the word location (top or bottom) and probe location (top or bottom) combinations to ensure that the probes replaced each of the words in each pair equally. The lexical probe task was completed once during the initial study visit.

#### Pictorial Probe Task

The pictorial task mirrored the lexical task, however, using pictures of food instead of words. All picture pairs were matched for ratings of valence and arousal (the rating based on results of pilot study by Kemps et al., 2014), as well as perceptual characteristics such as brightness and complexity. Unlike the lexical task, picture pairs were presented on either side of the central position. Participants were asked to place their left index finger on the “E” (to signal if the probe was on the left) key, and their right index finger on the “I” key (to signal if the probe was on the right), and to indicate as quickly as possible whether the probe replaced the right or left image. Intertrial interval and picture presentation time mirrored that of the lexical task. This pictorial task was completed once during the initial study visit and then repeated a second time following the field-based monitoring portion of the study.

#### Hunger Scales

To ensure participants had complied with instructions to eat a light meal up to 2 h prior testing and were satiated upon arrival, a modified version of the hunger scale (Castellanos et al., 2009; Loeber et al., 2012) was administered. Only the two relevant subscales measuring time since last eaten (an estimate to the nearest 15 min), and current level of hunger (rated on a sliding scale from 100 = *Not hungry at all* to 100 = *Extremely hungry*) were included.

## Results

In accordance with standard protocols (Kemps et al., 2014), mean reaction times (RTs) for critical (food—animal) trials were calculated after deletion of incorrect responses and outliers (i.e., RTs < 150 ms or > 1,500 ms, or RTs exceeding the individual’s mean + three standard deviations) for both the lexical and pictorial tasks. This resulted in deletion of 1.06% of the trials for the lexical task, and 1.03% (session 1) and 1.04% (session 2) of trials for the pictorial tasks. Control (animal—animal) trials were also discarded. An attentional bias score was calculated for each participant in three ways: “all food” (RT_animal_—RT_all food_) “high-caloric” (RT_animal_—RT_high__–__calorie food_) and “low-caloric” (RT_animal_—RT_low__–__calorie food_). For all three calculations, positive values indicated attentional bias toward food related stimuli. Mean attentional bias scores for both tasks and each of the different stimulus types are shown in Table 1 and the reaction times are shown in Table 2.

**TABLE 1:** Attentional Bias scores for all tasks, by stimulus category.

	**All food**	**High-caloric food**	**Low-caloric food**
	**Mean (*SD*) [Range]**	**Mean (*SD*) [Range]**	**Mean (*SD*) [Range]**
Lexical task	0.42 (18.27) [−37.69 −70.66]	0.72 (21.40) [−65.88 −72.89]	0.21 (22.02) [−52.49 −68.67]
Pictorial task 1	5.73 (19.29) [−33.94 −80.05]	5.27 (22.04) [−51.51 −65.25]	6.16 (21.52) [−32.32 −94.07]
Pictorial task 2	4.66 (14.72) [−28.10 −42.29]	4.39 (16.62) [−15.96 −43.93]	4.87 (17.99) [−34.85 −56.45]

*All food (RT_animal_—RT_*food*_), high-caloric (RT_animal_—RT_high–caloric food_) and low-caloric (RT_animal_—RT_low–caloric food_).*

**TABLE 2:** Mean reaction time for all tasks, by stimulus category.

	**All food**	**Animal**	***p***	**High-cal food**	**Low-cal food**	***p***
	**M (*SD*)**	**M (*SD*)**		**M (*SD*)**	**M (*SD*)**	
Lexical task	426.01 (75.82)	426.43 (73.72)	0.848	426.20 (76.32)	426.14 (72.88)	0.855
Pictorial task 1	411.30 (76.36)	417.02 (77.78)	0.016*	411.75 (76.03)	410.86 (77.98)	0.716
Pictorial task 2	386.00 (59.86)	390.66 (61.35)	0.011*	386.27 (62.55)	385.79 (58.71)	0.826

*p < 0.05.

### Comparison of Attentional Bias Scores Obtained From Different Tasks and Scoring Methods

To address our first aim, we compared attentional bias scores obtained from our two different visual probe tasks (lexical and pictorial), and three common scoring methods (all food, high-caloric and low-caloric), using data gathered during the initial study visit. Table 3 shows the correlation matrix of attentional bias scores calculated during the initial study visit. The tasks and scoring methods produced significant variation in the measure of attentional bias obtained. Within task correlations were highest when compared to all foods for both the pictorial and lexical tasks. However, comparisons between lexical and pictorial tasks were weak regardless of which scoring method was used. Across participants, the average correlation across the six scores was 0.57. Comparing the two tasks, the three scorings of the pictorial probe task showed slightly higher agreement.

**TABLE 3:** Correlations between the lexical and pictorial task attentional bias scores, both measured in session 1.

	**Pictorial**	**Pictorial**	**Pictorial**	**Lexical**	**Lexical**	**Lexical**
	**All food**	**High-cal**	**Low-cal**	**All food**	**High-cal**	**Low-cal**
Pictorial: all food	1.00					
Pictorial: high-cal	0.89	1.00				
Pictorial: low cal	0.88	0.57	1.00			
Lexical: all food	0.20	0.12	0.24	1.00		
Lexical: high-cal	0.23	0.15	0.26	0.84	1.00	
Lexical: low-cal	0.10	0.05	0.13	0.85	0.42	1.00

*Pictorial, pictorial task; Lexical, lexical task; high-cal, high caloric food images; low-cal, low caloric food images.*

### Test-Retest Reliability of the Pictorial Probe Task

To address our second study aim, we compared attentional bias scores obtained from the pictorial probe task at the initial study visit to those obtained when the task was re-administered at the final study visit (∼14-days later). Again, we used the three different attentional bias scoring procedures for the task, yielding attentional bias scores for all food stimuli, high-caloric food stimuli and low-caloric food stimuli. Regardless of the scoring procedure used, the test-retest reliability of the task was poor (low-caloric: *r* = 0.41; high-caloric: *r* = 0.20; all food: *r* = 0.40). As can be seen in Table 2, above, participants demonstrated faster response times to both food [*t*(68) = 5.62, *p* < 0.001] and animal [*t*(68) = 5.82, *p* < 0.001] stimuli in session 2 compared to session 1. A paired samples *t*-test confirmed that hunger levels remained consistent across both sessions [Session 1: *M* = 26.83, Session 2: *M* = 23.39; *t*(69) = 1.02, *p* = 0.312].

## Discussion

The aim of the present study was to assess the reliability of the visual probe task. We compared attentional bias scores from two of the most commonly used tasks—lexical and pictorial visual probe—using three different scoring methods. Our second aim was to evaluate the test-retest reliability of the pictorial probe task. The correlation between the lexical and pictorial tasks was weak. These findings are consistent with previous research (Freijy et al., 2014), and suggest that task type influences outcome—possibly contributing to the mixed findings within the literature assessing attentional bias using the probe task. The pictorial task yielded a wider range of attentional bias scores, with faster RTs to food compared to animal stimuli. The lexical task yielded similar RTs to both food and animal stimuli. This is in line with the notion that cues presented in picture form are more easily recognized—a phenomenon known as the superiority effect (Shepard, 1967; Snodgrass et al., 1972), and suggests that pictorial stimuli may be more useful for capturing attentional engagement.

The test-retest reliability for the pictorial probe task was also poor, regardless of how the attentional bias scores were calculated. This is an important finding, given that the visual probe task is frequently used in measuring food-related attentional bias. Poor test-retest reliability for the visual probe task is in line with previous findings on attentional bias measures in threat/anxiety (for a review, see Schmukle, 2005; MacLeod et al., 2019), and alcohol (Christiansen et al., 2015) research. Aday and Carlson (2019) found that the correlations between test-retest in the first two testing sessions were low, but increased over repeated testing sessions. Additionally, the attentional bias indexes from the later sessions correlated more strongly with participants’ trait anxiety scores, suggesting that extended testing may not only improve reliability, but that participants need extensive experience with the tasks in order for such biases to emerge. It is important to note, however, that the task Aday and Carlson (2019) used included personally relevant threatening stimuli. It has been previously demonstrated that using personally relevant stimuli increases internal reliability. For example, Christiansen et al. (2015) found that attentional bias toward personalized alcohol-related stimuli was larger than attentional bias to general alcohol-related stimuli, and, increased the internal reliability of the visual probe task. Future work in this area may like to consider making the food-related visual probe task more personalized to each participant by assessing food preference prior to testing. Additionally, in line with threat research carried out by Aday and Carlson (2019), it may be worthwhile assessing food-related attentional bias over multiple sessions, and correlate these results to participants’ more stable trait characteristics such as eating styles (Newman et al., 2008). However, it is important to consider that this approach may increase the risk of inflating assessment reactivity—potentially altering an individual’s attentional response style (MacLeod et al., 2019).

When calculating different attentional bias scores, the low-caloric and all food attentional bias scores had higher test-retest reliability compared to the high-caloric food attentional bias score. This is partially in line with previous research which found that all food attentional bias scores had the highest test-retest reliability (van Ens et al., 2019). Given the differences in stimulus presentation times between the present study and van Ens et al. (2019), further research is required to determine the influence of high-vs. low-caloric images on the reliability of the visual probe task. It is also possible that the improved reliability observed with the all food measure was simply due to it having a greater number of trials.

The present findings are in contrast to a recent study which found high test-retest reliability of attentional bias for food using the visual probe task (van Ens et al., 2019). van Ens et al. (2019) reported acceptable test-retest results for all food (*r* = 0.835) and high-caloric food (*r* = 0.611) RT indices. It is possible that the improved reliability was due to the longer stimulus presentation time of 3,000 ms, as it has been suggested that longer presentation times can improve reliability of time-reaction tasks (Waechter et al., 2014). However, it is important to note that longer stimulus presentation time (such as that used by van Ens et al., 2019) reflects the maintenance of attention rather than automatic attentional engagement (Mogg et al., 2004; Nijs and Franken, 2012). Theoretical accounts (such as incentive sensitization theory; Berridge, 2009) regarding food-related attentional bias suggest that this bias is driven by an automatic processing of food-related cues, which is why shorter stimulus presentation times (500 ms) are more common—unless specifically examining sustained attention (for example, see Nijs et al., 2010b).

### Limitations

The task parameters used in the present study were based on previous research with a stimulus presentation time of 500 ms (Kemps et al., 2014). Although this presentation time is commonly used (for example, see Kemps and Tiggemann, 2009; Ahern et al., 2010; Calitri et al., 2010; Nijs et al., 2010b; Kakoschke et al., 2015; Meule and Platte, 2016), using only the one presentation time is a limitation of the present study. While this presentation time has been used to measure “initial orientation” (Calitri et al., 2010), it has been suggested that attentional orienting occurs anywhere between 30 and 500 ms, disagreement at 500–1,000 ms, and avoidance at presentation times above 1,000 ms (Ouimet et al., 2009). Therefore, 500 ms presentation time could be tapping into either attentional orienting or disengagement. It is possible that during the 500 ms presentation time where two images are presented simultaneously, that multiple shifts of attention (i.e., attentional disengagement, shift, and engagement with new object) may occur (Doolan et al., 2015). As such, it has been argued that 500 ms presentation time does not reflect automatic orientation, but rather represents the cost of information processing by the attentional control mechanism (Starzomska, 2017). It has therefore been suggested that only very short presentation times (<500 ms) can provide insight into initial orientation of attention (Starzomska, 2017). Future studies could compare test-retest of both <500 ms and >500 ms presentation time to see which of these attentional processes may be more stable. Moving on, when examining the relationship between the lexical and pictorial probe tasks, it is important to note that although there was a high level of comparability between words and images, the stimuli were not 100% identical. Future studies may want to ensure that the stimuli are identical across tasks to minimize any confounding variables.

Contrary to previous findings (e.g., Doolan et al., 2015), participants were generally faster at responding to probes replacing low-caloric food items (i.e., fruits, vegetables, salads) than high-caloric food items (i.e., brownie, waffle, chips) at both testing sessions (see Table 2). This highlights another potential limitation; the current sample was made up of healthy participants with low/non-existent rates of disordered eating. Given that we expect food-related attentional bias to work in similar ways to alcohol-related attentional bias (i.e., based on theoretical models underlying addiction), it is possible that food-related attentional bias is more prevalent in those with pathological eating habits. Attentional bias scores may have higher reliability with individuals with underling eating pathology (who in turn are more likely to demonstrate higher levels of attentional bias toward palatable foods), as a higher range of true scores results in higher reliability (Waechter et al., 2014). However, studies assessing attentional bias have found increased attentional bias toward food in healthy individuals with obesity (i.e., Nijs et al., 2010b; Kakoschke et al., 2014; Kemps et al., 2014). As such, we should still expect that the attentional bias score obtained would be consistent across measures (particularly given that two thirds of the present sample were made up of individuals with overweight and obesity); something that we did not observe in our study. Also, findings suggest that variables such as affect and self-exertion also impact attentional bias toward food-related cues (Frayn et al., 2016; Pollert and Veilleux, 2018). It is plausible that some of these variables may have affected task performance between the two testing sessions. However, it is unlikely that such states would have varied enough between testing sessions to account for the poor test-retest reliability observed. Nonetheless, future studies should consider measuring and controlling for such state-like variables when assessing test-retest reliability. It is important to note that the effect of state-like variables on food-related attentional bias challenge the theoretical underpinning of attentional biases, which suggests that attentional bias should be relatively stable. This contrast between underlying theory and published findings on the effects of differing variables on attentional bias require closer examination.

The fact that this is a secondary analysis of a larger study examining real-world eating patterns also leads to limitations. As part of the larger study, participants underwent an intensive ∼14 day monitoring period during which they recorded all food and drink intake. It is possible that the monitoring may have influenced participants’ performance at the final attentional bias assessment. Table 2 shows that participants did generally have faster response times in session 2. However, given that response times shortened for both food and animal stimuli, this highlights the possibility that performance may have been affected by practice effects rather than cue reactivity. Another possible limitation regarding practice effects is that the participants were presented with the lexical and pictorial tasks in the same order. This lack of counterbalancing may have influenced response times. As the lexical probe task was always completed second, fatigue may have also influenced performance on the lexical task. Future research should replicate these findings using a counterbalanced design. Additionally, it is possible this 14-day period may have affected the results in other ways. It may be useful for future work to compare task performance following shorter periods to get a clearer picture of the effect of time on task performance.

## Conclusion

To conclude, the present study found correlations between the lexical and pictorial probe tasks to be weak. Furthermore, the test-retest reliability of the pictorial task was poor—regardless of how the attentional bias scores were calculated. Going forward, alternate measures of attentional bias should be explored [e.g., electrophysiological monitoring; findings suggest that event-related potentials capturing early attentional engagement have good reliability (Hagan et al., 2020)]. Finally, for attentional bias measures to be of any practical use, it would be useful to assess whether attentional bias is associated with real-world eating patterns.

## Data Availability Statement

The full dataset and the code book can be accessed here: https://rdp.utas.edu.au/metadata/2c3122be-fc62-48d0-a42d-41875d21e71b.

## Ethics Statement

The studies involving human participants were reviewed and approved by Tasmanian Social Sciences Human Research Ethics Committee. The patients/participants provided their written informed consent to participate in this study.

## Author Contributions

SFr and SFe developed the study concept and contributed to the study design. SFr and AM collected the data. SFr performed the data analysis and interpretation under the supervision of SFe. SFr drafted the manuscript. All authors provided revisions and approved the final version of the manuscript for submission.

## Conflict of Interest

The authors declare that the research was conducted in the absence of any commercial or financial relationships that could be construed as a potential conflict of interest.

## Publisher’s Note

All claims expressed in this article are solely those of the authors and do not necessarily represent those of their affiliated organizations, or those of the publisher, the editors and the reviewers. Any product that may be evaluated in this article, or claim that may be made by its manufacturer, is not guaranteed or endorsed by the publisher.

## Footnotes

1
https://osf.io/b9t2h

2
https://www.menzies.utas.edu.au/research/the-clinical-research-facility
